# Plantlet Anatomy of Silver Birch (*Betula pendula* Roth.) and Hybrid Aspen (*Populus tremuloides* Michx. × *Populus tremula* L.) Shows Intraspecific Reactions to Illumination *In Vitro*

**DOI:** 10.3390/plants11081097

**Published:** 2022-04-18

**Authors:** Mārtiņš Zeps, Toms Kondratovičs, Elva Grigžde, Āris Jansons, Pauls Zeltiņš, Ineta Samsone, Roberts Matisons

**Affiliations:** Latvian State Forest Research Institute “SILAVA”, Rīgas Street 111, LV-2169 Salaspils, Latvia; toms.kondratovis@silava.lv (T.K.); egrigzde@inbox.lv (E.G.); aris.jansons@silava.lv (Ā.J.); pauls.zeltins@silava.lv (P.Z.); ineta.samsone@silava.lv (I.S.); roberts.matisons@silava.lv (R.M.)

**Keywords:** micropropagation, clones, ex vitro, stomata density, xylem and phloem

## Abstract

Micropropagation of forest reproductive material is becoming an increasingly important tool of climate-smart forest management, whose efficiency is depending on artificial illumination, which in turn can have species-specific effects. To improve the energy-efficiency of micropropagation, light emitting diodes (LED) are becoming more popular; however, they emit light of narrow spectral composition, synergic effects of which can alter plantlet development. Regarding the in vitro cultures of trees, such effects have been scarcely studied. In this study, three clones of silver birch (*Betula pendula* Roth.) and three clones of hybrid aspen (*Populus tremuloides* Michx. × *Populus tremula* L.) from the eastern Baltic region were tested. The responses of leaf and stem anatomy of in vitro cultures to three LED light illumination treatments differing by spectral composition and to illumination by fluorescent tubes were estimated by linear (mixed) models. The studied light treatments had non-interacted effects on stomata density and on the secondary xylem cell wall in the stem of silver birch and in the stomata length, stem radius, and phloem width of hybrid aspen. Furthermore, clone-specific responses to illumination were observed for number of chloroplasts and phloem width of silver birch and for leaf thickness and xylem cell wall thickness of hybrid aspen, implying different mechanisms of shade avoidance. In general, the responses of plantlet anatomy differed according to the width of the light spectrum in case of LED, as well as for fluorescent tubes. Considering the legacy effects of early development of plantlets, adaptability of illumination in terms of spectral composition according to the requirements of genotypes appear highly beneficial for micropropagation of sustainable forest reproductive material.

## 1. Introduction

The accelerating climatic changes are highlighting the necessity for climate-smart forest management, which puts emphasis on a wider application of the most sustainable and productive plant reproductive material [1]. Considering the growing environmental risks for conventional cultivation [2], in vitro micropropagation is becoming a more reasonable source of forest reproductive material. This process, however, is equipment demanding and energy intensive compared to conventional plant propagation, as artificial illumination is needed [3]. In this regard, light emitting diodes (LED) are gaining increasing application due to their long lifespan, superior electric efficiency, compact size, and reduced thermal radiation [4,5,6]. However, LED emits light of narrow spectral composition; hence, effort should be paid for research of optimal spectral compositions, which would be optimized regarding the requirements of specific plant material [4,7,8,9].

Beside the differences in photosynthetic efficiency, light spectrum also acts as an environmental signal allowing plants to assess the surrounding environment and to adjust their physiology and morphology accordingly [9]. Such signaling effects are particularly explicit under controlled environments of in vitro cultures [10,11]. Light stimuli are captured by several photoreceptors, which can trigger reactions adjusting the state of a plant [12,13,14]. Phytochromes are generally sensitive to the red (R) and far red (FR) spectrum [15] affecting the development of stomata and leaf movement [16,17,18]. Reactions to increased R radiation also include higher biomass production [18,19,20] and activation of antioxidant systems [21], as a response to high light availability [22]. However, monochromatic (R) light reduces photosynthetic capacity and inhibits growth [23]. Cryptochromes are sensitive to blue and UV-A and are involved in regulation of relatively higher chlorophyll concentration, larger leaf area [22,24,25,26]. Phototropins, which regulate leaf and chloroplast movement, are also sensitive to blue (B) and UV-A light [27].

Evolutionary plants have adapted to function under the full light spectrum, and the responses to the monochromatic light, which are observed under lab conditions, can be misleading, as the intrinsic signals interact [6,14,18,25,28]. Such interactions are essential for efficient and controlled shade avoidance and adjustments of physiology according to canopy status [29,30]. The red-blue (R:B) ratio is the main light characteristic regulating the majority of physiological processes in dicot plants, which subsequently are resulting in alterations in anatomy and morphology [18,28]. The combination of B and R light controls biomass formation, number and thickness of leaves, as well as chlorophyll concentration and stomata density and size, and such reactions cannot be observed under monochromatic lights [18,31,32,33]. Furthermore, green (G) light, which penetrates the canopy better than others facilitating the assimilation of CO_2_ in shaded parts of the crown [32,33,34], can modulate the signaling effects of the R:B ratio, facilitating physiological and anatomical adaptation to shade [35].

The anatomy of the leaf and stem of plantlets, which is affected by spectral composition of light and substrate, is crucial for micropropagation of reproductive material [35,36,37,38,39]. Although the anatomy of leaves express high plasticity in response to light conditions, which is easy to observe [40], there are also the legacy effects of light conditions, which can substantially alter the functioning and further development of a plantlet later in ex vitro conditions [16,41,42]. For instance, increased number and/or size of stomata can subject plantlets to drought stress, if the atmospheric water demand increases and roots have not formed sufficiently [6,17,18,34]. Alterations in the number of chloroplasts can affect assimilation after a transfer to another light condition [26].

From the practical point of view, the anatomy of the stem is substantial for manipulations in vitro, as well as for further development of a plantlet ex vitro [3,6]. Larger stem diameter is crucial for convenience of manipulation of plantlets in vitro and subsequent transfer to ex vitro [3]. Width and anatomy of the xylem can play a crucial role when water transfer intensity shifts when plantlets are transferred to ex vitro [3,43,44], while phloem width represents the nutrient reserves needed for early development [3,6]. Accordingly, light conditions during the in vitro cultivation phase can significantly affect the entire propagation process.

Although the main responses of plants to light spectral composition have been identified, there is a high variability among taxa, and even populations, due to local adaptation of plants and, particularly, trees [4,7,8,9,16,45]. Accordingly, species- or population-specific information might be necessary to optimize the efficiency of in vitro propagation [9,45]. Furthermore, most of the experiments have been made on model plants, such as *Arabidopsis* or widely cultivated species, such as tomatoes and potatoes (*Solanum* spp.), while trees have received considerably less attention [6]. Accordingly, the aim of the study was to evaluate the responses of the anatomy of plantlets of clones of silver birch (*Betula pendula*) and hybrid aspen (*Populus tremuloides* × *P. tremula*) to artificial illumination of differing spectral composition in vitro. Such genotypes were selected as they are commercially propagated by in vitro methods in Northern Europe due to the growing economic interest in establishing productive and sustainable stands. We hypothesized that the responses of leaf anatomy to light conditions would be “species”-specific due to differing early growth strategies. We also assume that silver birch as light demanding species would be more sensitive to the illumination containing increased amount of R light, while hybrid aspen, which is shade tolerant at young age, to increased levels of B and G light.

## 2. Results

The anatomical variables (proxies) exhibited different degrees of variation between silver birch and hybrid aspen. For silver birch, the highest variation (coefficient of) was estimated for stem radius, xylem and phloem widths, and secondary xylem cell wall thickness (SXCWT) (0.43–0.55). The variation of stomata density, stomata width and length, leaf thickness, and number of chloroplasts was considerably lower (0.10–0.17) (Table 1). Low variation of all measured anatomical proxies was estimated for hybrid aspen, with the coefficient of variation ranging 0.09–0.23 for stem radius and stomata density, respectively.

The linkage among the anatomical proxies differed by species and also by clones, as indicated by linear Pearson correlations between them, indicating diverse regulatory mechanisms. Silver birch showed stronger correlations compared to those of hybrid aspen. Nevertheless, most of the proxies showed weak to moderate correlations, while the highest correlations were generally observed between stem radius and widths phloem and xylem (Table S2). Clone-specific correlations were observed among anatomical proxies of leaves.

The studied anatomical proxies varied greatly among clones; nevertheless, the studied light treatments had significant effects on the anatomy of silver birch and hybrid aspen in vitro culture (Table 1). However, the effects of light treatments were species- and clone-specific, indicating high variability of responses, thus highlighting the necessity for individual approaches. For silver birch, the studied light treatments had significant non-interacted effects on SXCWT and, particularly, on the stomata density (Table 1). In turn, the number of chloroplasts and, particularly, the phloem width were affected by the interaction of light treatment and clone, indicating complex relationships (Table 1).

**Figure 1:**
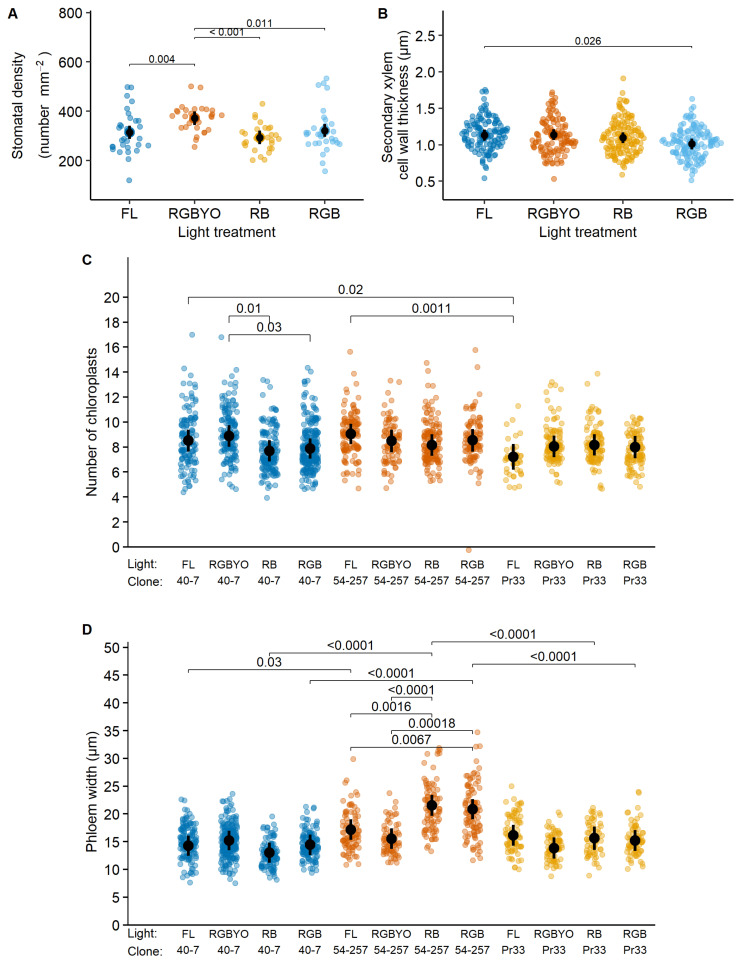
Response to light of stomata density (**A**), second xylem cell wall width thickness (**B**), response to clone by light interaction of number of chloroplast (**C**), phloem width (**D**) of silver birch clones in vitro shoots cultured under fluorescent (FL) light and different light spectrum LED light (RGBYO, RB, RGB). Mean values with 95% confidence intervals shown in black; significant *p*-values for multiple comparisons of all light treatments using Tukey HSD post hoc test shown above denoting statistically significant differences (*p* ≤ 0.05). Dots in colors denote data points used for analysis.

The SXCWT of silver birch was reduced under the luminaire that contained R, G, and B LED light (RGB); however, the pairwise differences were significant only in the case of the strongest contrast between fluorescent luminaire (FL) and LED luminaire who contained R, G, B, yellow (Y), and orange (O) light (RGBYO) (Figure 1B), indicating sensitivity to Y and O parts of light spectrum. In contrast, stomata density increased under RGBYO, which simultaneously contained R, Y, and O parts of light spectrum (Table 2), compared to other treatments (Figure 1A). Stomata density lacked significant pairwise differences between other treatments, irrespectively of R:B and red:far-red (R:FR) ratio (Table 2), thus supporting its sensitivity to interacting signals of specific parts of light spectrum.

The significant light by clone interaction for the number of chloroplasts of silver birch was apparently caused by significantly higher values estimated for clone No. 40-7 under the RGBYO treatment compared to RB and RGB (Figure 1C), showing to sensitivity to Y and O part of light spectrum. Regarding phloem width, the more productive clone No. 54-257 showed increased values under LED luminaires that contained R and B (RB) and R, B, and G (RGB) light spectrum part, which lacked the Y and O parts of light spectrum. The differences among other clone-light combinations were non-significant, although the less productive clones No. Pr33 under RGBYO and No. 40-7 under RGB and FL treatments tended to form narrower phloem (Figure 1D), suggesting an opposite reaction. 

**Figure 2:**
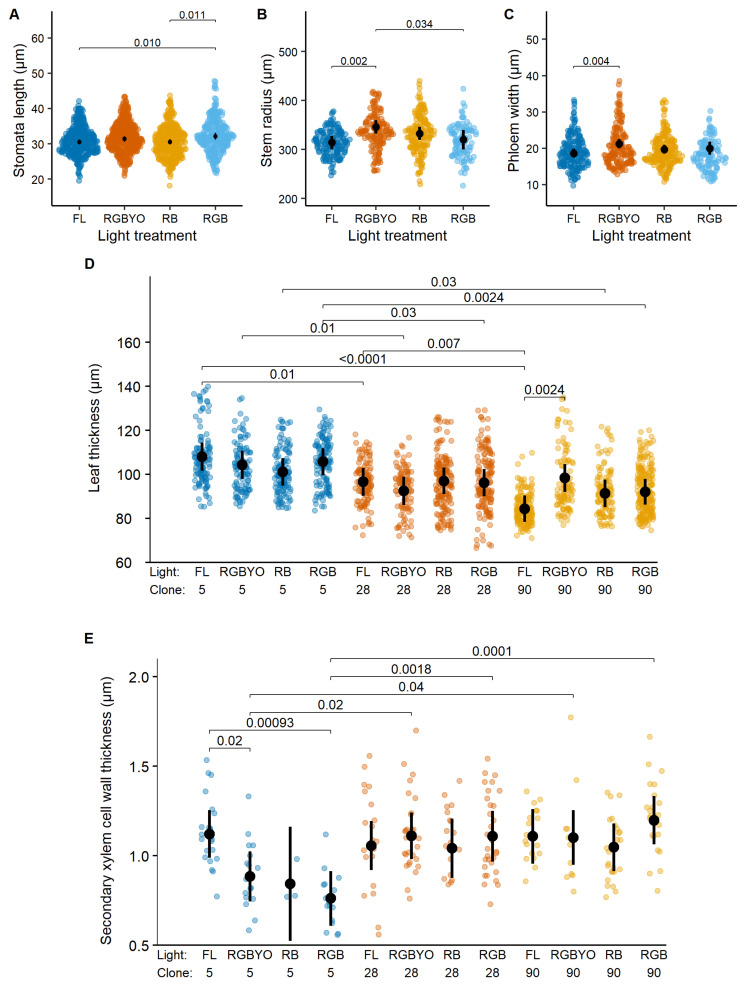
Response to light of stomata length (**A**), stem radius (**B**), phloem width (**C**), response to clone by light interaction of leaf thickness (**D**), and secondary xylem cell wall thickness (**E**) of hybrid aspen clones in vitro shoots cultured under fluorescent (FL) light and different light spectrum LED light (RGBYO, RB, RGB). Mean values with 95% confidence intervals shown in black; significant *p*-values for multiple comparisons of all light treatments using Tukey HSD post hoc test shown above denoting statistically significant differences (*p* ≤ 0.05). Dots in color denote data points used for analysis.

Stomata length, stem radius, and phloem width of hybrid aspen showed significant individual effects of light treatment, while leaf thickness and SXCWT showed clone-specific responses to illumination (Table 1). Furthermore, the clone-specific responses of phloem width and SXCWT for hybrid aspen to light treatments were opposite to those two observed for silver birch. The stomata length increased under RGB treatment; however, the pairwise differences were significant only for the strongest contrast (RB vs. FL) (Figure 2A), suggesting a stimulating effect of illumination containing G and R parts of the light spectrum. Also, in the case of RGBYO, stomata length tended to be higher. The RGBYO treatment, which exceled by B and G light ratio (Table 2), facilitated radial growth of hybrid aspen plantlets, as indicated by significantly higher stem diameter compared to treatments with lower amounts of B light (Figure 2B). However, phloem width, which is a fraction of stem radius, was reduced under FL treatment compared to RGBYO, which showed the highest value (Figure 2C). Such response was contrasting to silver birch, for which RB and RGB treatments were stimulating, although clone-specifically (Figure 1D).

The effect of clone by light interaction on SXCWT of hybrid aspen apparently was caused by the negative responses of the less productive clone No. 5 to LED light treatments RGBYO, RGB, and, particularly, RB (Figure 2E), while the differences between other combinations were non-significant. Such response partially mimicked that of silver birch, which responded negatively to RGB (Figure 1B). Furthermore, clone-specific responses of hybrid aspen to light treatments were explicit for the leaf thickness (Figure 2D). A more productive clone No. 90 reduced leaf thickness under FL treatment compared to RGBYO, which showed the highest value (Figure 2D), indicating a reaction to the amount of R light in the total spectrum. The differences among other clone-light combinations were non-significant, although the less productive clones No. 5 under RB and No. 28 under RGBYO treatments tended to form thinner leaves (Figure 2D), suggesting an opposite reaction.

## 3. Discussion

The responses of the anatomical proxies to studied light treatments (Table 1) supported different early growth strategies of silver birch and hybrid aspen, which in the case of hybrid aspen, could also be related to heterosis [46]. This implies that “species” or even clone-specific adjustments in illumination can optimize in vitro propagation of forest reproductive material of silver birch and hybrid aspen and improve its quality, thus contributing to climate-smart forestry [2]. The initial conditions during early development are known to result in considerable long-term effects for the whole propagation process [47,48,49], supporting the importance of cultivation conditions of plantlets [3,49].

During the propagation, plantlet stems are repeatedly injured by cutting and by insertion into growing media both in vitro and ex vitro [3,48], hence thicker and more robust stems are favorable to reduce damages [48,49]. Also, thicker plantlet stems allow more convenient manipulations, as they are less fragile. Directly after a transfer, the plantlet stem is a substantial source of nutrient reserves, which are utilized for acclimation to novel growing media [6], healing the physical damage, and early development of a root system [49]. Accordingly, thickness of stem and particularly phloem is a crucial property, which is indicative of the ability of plantlet to acclimate [50,51]. Also, xylem anatomy determines the sensitivity of hydraulic architecture of the plantlet to environmental fluctuations [52,53], which during the propagation process mainly arise from transfers [49].

The main function of the xylem is water (sap) transport to compensate transpiration [18,50], which in vitro, however, is low due to a highly saturated atmosphere [28,54]. Accordingly, one can assume that in vitro, the xylem functions optimally, irrespectively of its properties, as suggested by the lack of significant relationships between stomata density and xylem properties of birch and hybrid aspen clones (Table S1). Although light composition has been shown to affect xylem width in herbaceous plants [31,43], such effects were lacking for plantlets of silver birch and hybrid aspen, suggesting specifics of responses related to the type of life form [43,55]. The effect of illumination might be indirect via facilitation of biomass accumulation under increased blue illumination [56,57], as hinted by the correlation between widths of xylem and phloem and stem diameter for birch and hybrid aspen. The correlations, however, support the differences in radial early growth strategies between the “species” with birch increasing phloem irrespectively of xylem width (Figure 1D, Figure 2C and Table S1).

Phloem width, is relatively stable irrespectively of plant height [51], thus suggesting potential of intrinsic nutrient reallocation crucial for regenerated after damage [58,59]. Leaf gas exchange is related to transport of assimilates through phloem [60], supporting the complex response of phloem width to R, Y, and O light (Figure 1D and Figure 2C), which generally facilitate efficiency of photosynthesis [14,16,18,20]. The SXCWT, which provides the mechanical strength [61,62], responded similarly to phloem width (Figure 1B). During xylogenesis, cell wall thickness is adjusted to meet current water relations of a plant [63] via optimization of conductivity and intrinsic carbon budget [64,65]. Although significant, the responses of SXCWT to light treatments were quite small under controlled optimum conditions, still, their influence might be amplified during the ex vitro phase, when functionality of xylem increases.

In contrast to birch, aspen can be more shade tolerant at a young age [66,67], implying different mechanisms of shade avoidance [30,66]. The amount of R and FR, B and UV-A light, as well as the B:G light ratio are the main signals of shade conditions, which can be species-specific [27,30,68,69,70,71]. The responsiveness of hybrid aspen to G light (Figure 2B) might be related to the ability of both parental species to regenerate by root suckers, even at extreme densities [72]. Apparently, these suckers are sensitive to G light, which penetrates deeper layers of the canopy, and in combination with B light, triggers shade avoidance [35,73]. The reduction of the stem diameter of hybrid aspen (Figure 2B) under the light treatments with low B:G ratio (FL and RGB; Table 2) suggested that increased G illumination triggered shade avoidance of plantlets [35,73], particularly as plantlet height was unaffected by illumination [74]. For silver birch, shade avoidance was apparently triggered by a decreased R:FR ratio [70,71], which in all of the experimental treatments was high (Table 2), thus explaining the absence of differences in plantlet stem diameter (Table S1).

Leaf anatomy is highly plastic in terms of responses to light conditions [39], which, however, can have substantial legacy effects on further development of a plantlet via alterations of photosynthetic apparatus and, particularly, stomata density and chloroplast number [73]. Accordingly, the ability to adjust leaves to a relevant stage of the micropropagation, particularly, ex vitro phase, can improve plantlet performance [49]. The ability to adjust stomata characteristics is also considered as a proxy for adaptability of genotypes [75], which appeared higher for birch irrespectively of the clone (Table 1). Considering that stomatal characteristics have been related to the intensity of full sun light spectrum [19,40,41], effects of which can differ among species [40,76,77], the significant effects of light treatments indicated sensitivity of stomata anatomy and density to spectral compositions of light (Figure 1A and Figure 2A). For instance, the development of stomata is facilitated by the signals of phytochromes in response to increased R and FR radiation [78]. Low R:FR ratio decreased stomata density in *A. thaliana*, *Citrus insitorum* and *Oryza sativa* [79,80,81], while for others, such response might be lacking [82] or positive effect of the B light can occur [18,80,83]. However, most of these studies have focused on the responses to monochromatic light, which might be misleading [5,6]. For both birch and hybrid aspen, R:FR ratio of experimental light treatments, which substantially exceeded natural (<1, [84,85]) (Table 2), did not affect stomata density. However, stomata density increased under extended light spectrum (RGBYO) for silver birch clones, suggesting explicit reaction to open canopy conditions [36]. From the practical point of view, increased stomata density can have dual effect. Increased stomata density might facilitate further development ex vitro due to improve gas exchange in leaves [18,54]. On the other hand, increased stomata density might subject plantlets to increased transpiration, if atmospheric water demands increase [54,86].

In contrast to silver birch, hybrid aspen adjusted stomata length, while maintaining the same density (Table 2). Increased stomata length improved conductivity; however, did not affect reaction speed [86], thus allowing higher plasticity of responses to changes in atmospheric water demand [87]. Longer stomata under R light and G light, increased potential efficiency of gas exchange and transpiration, which can have a critical role both in in vitro and ex vitro. The responses to light conditions were complex, as lack of G light decreases CO_2_ assimilation [88], while decreased intensity of R light affects development of stomata [29].

Chloroplast count, which affects the rate of assimilation [18,38,73] was clone-specifically affected by the light treatments (Table 1), supporting local adaptation of birch provenances to open canopy conditions. For clone No. 40-7, chloroplast count was sensitive to the Y and O light (Figure 1C). Still, chlorophyll concertation, which is a complementary proxy was not assessed [26]. Leaf thickness, which is strongly subjected to legacy effect of preceding growing conditions [40], for hybrid aspen, clone No. 90 was sensitive to the quantity of R light (Figure 2D). B and R light is mostly absorbed by the chloroplasts located in the upper part of a leaf, while G light penetrates deeper [89]. Accordingly, R and G light might be expected to have the strongest effect on leaf anatomy [20,35], which was not the case in this study, probably due to decreased leaf thickness under in vitro conditions [28]. The number of chloroplasts is indicative of potential photosynthetic capacity [26,38], while leaf thickness suggests vitality of leaf and its robustness against the manipulations during the propagation process.

## 4. Materials and Methods

### 4.1. Experimental Setup

To evaluate the responses of silver birch and hybrid aspen clones in vitro cultures to illumination, four light treatments were tested under controlled conditions. The tests were performed in a climatic chamber, where 25 °C temperature and 30–40% relative humidity were maintained. Within the chamber, four multi-store shelving systems with a shelf size of 120 × 100 cm and shelf height of 35 cm were placed. Each shelf was equipped with luminaries placed 30 cm above the shelf surface. Non-transparent screens were placed between the shelving systems to avoid light contamination from other treatments.

**Figure 3:**
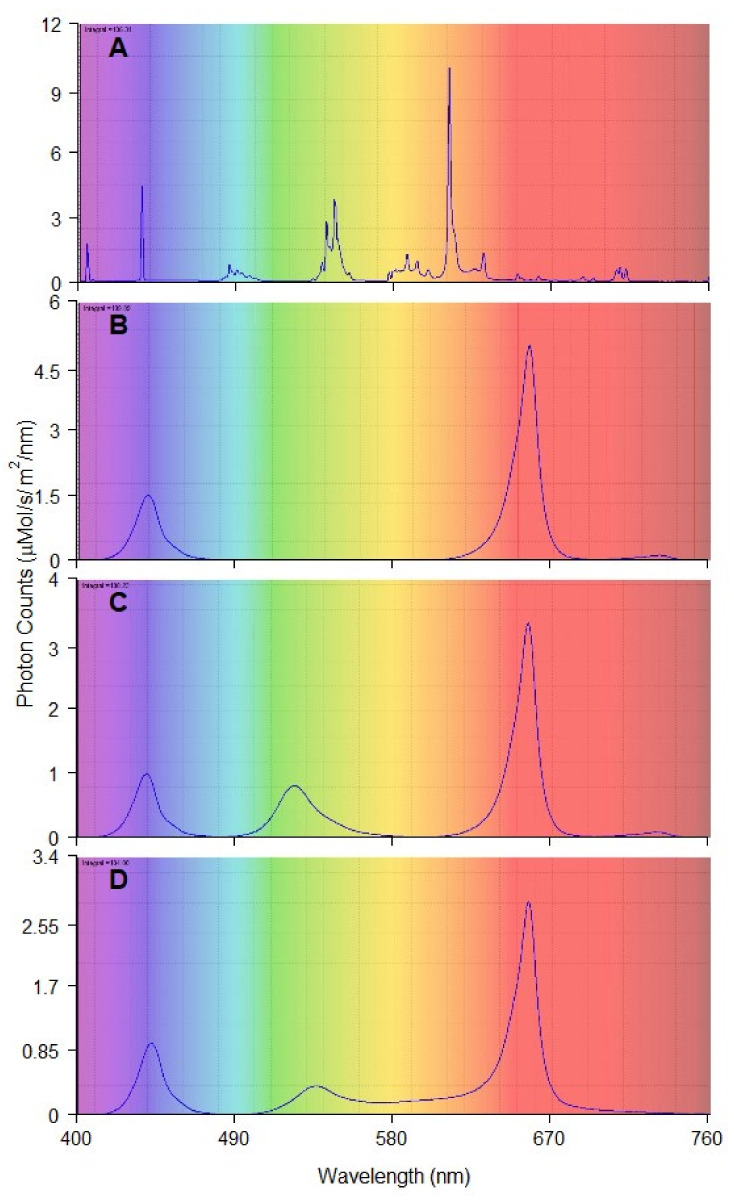
Spectral composition and photon count of each lighting treatment: (**A**) fluorescent tubes (FL); (**B**) Red and Blue (RB), max photon count at 655 and 440 nm; (**C**) Red, Green and Blue (RGB) max photon count at 655, 520, 440 nm; (**D**) Red, Green, Orange, Blue (RGBYO), max photon count 655, 535, 625, 445 nm.

The tested light treatments were a combination of (1) red and blue LED light (RB); (2) red, green, and blue LED light (RGB); (3) red, green, blue, yellow, and orange LED light (RGBYO) (Figure 3). Such combinations were used to test the synergic effects of the parts of spectrum with signaling effects on different photoreceptors, as well as on the photosynthetic efficiency [5,14,17,18,57]. Light from conveniently used fluorescent tubes Philips Master TL-D 36W warm white was used as the control. Additionally, far-red (FR) diodes were incorporated in the RB and RGB (LED) treatments to provide a spectral region of phytochrome absorbance [79,80]. In RGBYO treatment, FR spectrum was provided by yellow diode, which emits a broader spectrum of light. All LED light treatments had a red:blue (R:B) ratio of 3.2:1 and red:far-red (R:FR) ratio range of 28–36:1. The fluorescent light (FL) had R:B ratio of approximately 0.24:1 and R:FR of 3:1 (Table 2), thus distinguishing it from others.

The photon flux density of 110 ± 10 µmol m^−2^ s^−1^ (range 400 to 750 nm) for all light treatments and 16/8 h light/dark photoperiod was maintained. To ensure the uniformity of illumination intensity, each shelf was divided into 100 cm^2^ squares, and the illuminance spectrum and intensity were verified for each square using AvaSpec ULS2048 spectrometer (Avantes, Apeldoorn, The Netherlands). Adjustments of intensities of the illumination were made if necessary.

### 4.2. Plant Material

Silver birch was represented by three clones of open-pollinated progenies of plus-trees from the eastern part of Latvia (55°40′–58°05′ N, 20°58′–28°14′ E) [90], which were obtained from a progeny trial in the central part of Latvia (56°44′ N, 24°49′ E). Studied silver birch provenance is known for high-quality trees [91], and the trial was established under the national breeding program [90]. The studied clones were selected according to their field performance, ranging from intermediate to superior for clones No. Pr33, No. 40-7, and No. 54-257, respectively. The material of hybrid aspen was obtained from the progenies of controlled crossing of plus-trees of local common aspen (*Populus tremula*) and American aspen (*Populus tremuloides*) growing in a botanical garden in the central part of Latvia. Three clones of progenies were selected based on their field performance within the trial; clone No. 5 represented the less productive genotypes with the field performance below the native common aspen population. Clone No. 28 had an intermediate field performance, which slightly exceeded the natural population of common aspen; while clone No. 90 showed superior productivity. The plant material was collected from a trial in the central part of Latvia (56°44′ N, 24°49′ E).

Prior to the exposition to light treatments, the plant material had been maintained within in vitro clone collection in the plant physiology laboratory of LSFRI Silava approximately for five years. In the clone collection, birch plantlets were cultivated on woody plant medium (WPM) [92], supplemented with WPM micronutrients, WPM vitamins, 0.1 mg L^−1^ zeatin, 20 g L^−1^ of sucrose, and 6 g L^−1^ agar (Sigma-Aldrich, St. Louis, MO, USA). The hybrid aspen plantlets were cultivated on ½ Murashige and Skoog medium (MS) [93], supplemented with MS micronutrients, MS vitamins, 0.1 mg L^−1^ idole-3-butyric acid (IBA), 20 g L^−1^ of sucrose, and 6 g L^−1^ agar (Sigma-Aldrich, St. Louis, MO, USA). The pH of the medium was adjusted to 5.8 before autoclaving for 15 min (110 kPa, 121 °C). All plantlets were growing under the same illumination provided by Philips Master TL-D 36W florescent tubes, with photon flux density of 110 ± 10 µmol m^−2^ s^−1^.

For both birch and hybrid aspen, ~1.5 cm plantlet apices were excised and transferred to 300 mL glass jars, each containing 30 mL of the relevant plant medium. Eight plantlets were inserted per jar and jars were sealed with aluminum foil. Twenty jars were prepared for each clone, five jars for each light treatment, respectively (120 jars and 960 plantlets in total). To evaluate the effect of the illumination treatments on the development of plantlets, the jars were placed under the experimental light treatments for 30 days. The jars containing birch and hybrid aspen were randomly distributed on the shelves with a 5 cm distance between them.

### 4.3. Measurements

To assess the anatomical responses to light treatments, two plantlets were randomly selected from each jar. For one plantlet, the second and third leaf from the apex were excised, and from each leaf, a random 3 × 2 mm fragment was cut for measurements of stomata. Older leaves were avoided to avoid the legacy effects of preceding conditions [41,94]. High-resolution (5184 × 3456 px) images from each fragment were acquired at 40× *g* magnification. Samples were submersed in water for 15 min prior acquisitions of images. The length and width of individual stomata, as well as the stomata density (number per 1 mm^2^) were measured from the image (Figures S1 and S2). At least 15 stomata for each sample were measured.

Another randomly selected plantlet from each jar was used for the measurements of chloroplast number in cell, leaf thickness, stem diameter, and width of xylem and phloem, as well as SXCWT. To measure chloroplast number, and leaf thickness, the second leaf from the apex was taken and 10–12 serial thin cross-sections (15–20 µm thick) were cut. The sectioning was done in the mid-part of the leaf using a GLS1 hand microtome (Schenkung Dapples, Zürich, Switzerland). High-resolution images from each fragment were acquired at 200× *g* magnification. The chloroplast number per cell was counted in three to four randomly selected parenchyma cells within each thin section (≥30 cells per leaf evaluated in total). For the measurements of leaf thickness, 40× *g* magnification images of the same thin sections were taken. Leaf thickness was measured for each cross-section image at an approximately 2 mm distance from the central vein, avoiding any secondary veins.

From the same plantlets, stem sections from the mid-part of the third internode part from the apex were taken, and for each of them, 10 serial thin sections were cut with the microtome. To increase the contrast between the xylem and phloem, double staining with Astrablue and safranin was performed according to Gärtner and Schweingruber [95]. The cross-sections were rinsed with water and 40, 70, and 96% ethanol. For measurements of stem diameter, thickness of xylem and phloem, cross-section images were acquired at 40× *g* magnification (Figures S3 and S4). For the measurements of SXCWT, images were acquired at 200× magnification.

All measurements were done in the ImageJ v1.8 software (Wayne Rasband, National Institutes of Health, USA). The “multipoint” function was used for the measurements of stomata density; the function “straight” was used to measure stem radius, thickness of leaf, phloem and xylem, SXCWT, stomata length and width. The SXCWT was measured for five cells per image; three measurements in random direction per cell were done. All images were acquired using Leica DM1000M (Leica microsystems, Wetzlar, Germany) transparent light microscope equipped with a SLR camera Canon EOS 4000D (Canon, Tokyo, Japan).

### 4.4. Statistical Analysis

Considering that silver birch and hybrid aspen were grown on different media, the data were analyzed separately for each “species”. To assess the linkage between the measured anatomical proxies, Pearson correlation analysis was conducted for each clone. The effects of light treatments and clone on the anatomy of plantlets were assessed using linear mixed-effects models or generalized linear mixed-effects models applying Poisson residual distribution according to data type analyzed.

The statistical models in the general form for stomata length and width was as follows:

Y_ijklm_ = μ + LED_i_ + C_j_ + LED_i_ × C_j_ + l_k_ + i_kl_ + ε_ijklm_, (1)

where Y_ijklm_ is the response variable, μ is the overall mean; LED_i_, C_j_, and LED_i_×C_j_ are the fixed effects of light treatment, clone, and the light treatment by clone interaction, respectively. The l_k_ and i_kl_ are the random effects—the measured leaf and separate images of the leaf, respectively, and ε_ijklm_ is the random error. For stomata density, fixed effects linear model in the same form was used (random effects excluded). 

For leaf thickness, stem radius, phloem width and xylem width, the following mixed models were used:

Y_ijklm_ = μ + LED_i_ + C_j_ + LED_i_C_j_ + s_k_ + i_kl_ + ε_ijklm_, (2)

where, s_k_ and i_kl_ are the random effects, the plantlets and separate images of the plantlets, respectively. For number of chloroplasts and SXWCT, the reduced forms of the Equations (1) and (2), respectively, without the random effect of separate image of leaf or plantlets, were used.

The models were fit using the restricted maximum likelihood approach. The estimated marginal means for the levels of significant effects were compared using the Tukey′s HSD multiple comparison test. The data analysis was performed in R v. 4.1.2. [96] using packages “lme4” [97] and “emmeans” [98].

## 5. Conclusions

Spectral composition of illumination had significant intra- and inter-specific effects on the anatomy of silver birch and hybrid aspen plantlets in vitro, likely as a result of local adaption and heterosis, respectively. Such effects were explicit under the studied LED light, which emits light of narrower spectral composition compared to conventionally used, yet less energy-efficient fluorescent tubes. However, the observed responses suggest that LED light can be combined to improve sustainability of the propagated plant material via alteration of their anatomy. Considering the legacy effects of early development, specific light sensitivity might lead to an uneven development of the plantlet, reducing the efficiency of micropropagation process, hinting the necessity for species- and even population-specific adjustments of illumination. Accordingly, plasticity of LED luminaries in terms of editing the composition of light appears highly advantageous for increasing efficiency of micropropagation of trees and forest reproductive material. Luminaries capable of emitting RGBYO light or wider are advised. Furthermore, the effects of light composition in terms of wider spectrum and intensity of different parts of the spectrum might provide additional options for improvements of propagation of plant material for specific conditions.

## Figures and Tables

**Table 1:** Different effects of spectral composition of light on leaf and stem anatomical properties.

	Silver Birch			Hybrid Aspen		
	Light	Clone	Light by Clone	Light	Clone	Light by Clone
Stomata density, µm	5.8 ***	0.8	1.2	2.5	29.9 ***	2.1
Stomata length, µm	1.2	25.1 ***	1.6	2.9 *	64.4 ***	1.8
Stomata width, µm	1.7	38.4 ***	1.6	2.2	19.2 ***	2.1
Leaf thickness, µm	0.6	0.4	0.4	0.4	28.5 ***	3.2 *
Number of chloroplasts	1.6	3.3 *	2.0 *	1.2	4.0	2.1
Stem radius, µm	0.2	101.6 ***	0.3	4.0 **	1.3	2.6
Xylem width, µm	0.9	49.9 ***	1.0	1.2	9.4 ***	0.5
Phloem width, µm	3.0 *	26.9 ***	4.6 ***	4.5 **	46.4 ***	1.2
Secondary xylem cell wall thickness, µm	2.6 *	7.6 ***	0.9	0.6	7.7 ***	2.3 *

* Significant at *p* < 0.05; ** significant at *p* < 0.01; *** significant at *p* < 0.001.

**Table 2:** Spectral composition % of total photon flux (from 400 to 750 nm) for light treatments used in this experiment.

	Red and Blue (RB)	Red and Green and Blue (RGB)	Red and Green and Blue and Yellow and Orange (RGBYO)	Fluorescent Tubes (FL)
Blue 400–500 nm	23	18	17	17
Green 500–570 nm	0	22	17	25
Yellow 570–590 nm	0	0	3	7
Orange 590–625 nm	2	1	5	36
Red 625–700 nm	73	57	56	11
Far-red 700–750 nm	2	2	2	4
Red:Blue (R:B)	3.17	3.17	3.29	0.65
Red:Far-red (R:FR)	36.5	28.5	28	2.75
Blue:Green (B:G)	n/a	0.82	1.00	0.68

## Data Availability

The datasets generated during and/or analyzed during the current study are available from the corresponding author on reasonable request.

## Supplementary Materials

The following supporting information can be downloaded at: https://www.mdpi.com/article/10.3390/plants11081097/s1, Table S1: Stomata parameters, chloroplast count, leaf thickness, and stem anatomical parameters of silver birch and hybrid aspen in vitro cultures grown under different spectral compositions, Table S2: Pearson correlation coefficients among anatomical proxies of hybrid aspen and silver birch clones across the studied light treatments at jar level, Figure S1: Stomata of silver birch plants grown under different light treatments, Figure S2: Stomata of hybrid aspen plants grown under different light treatments, Figure S3: Cross-sections of silver birch in vitro plants grown under different light treatments, Figure S4: Cross-sections of hybrid aspen in vitro plants grown under different light treatments.
