# Supplementary material for: Plantlet Anatomy of Silver Birch (Betula pendula Roth.) and Hybrid Aspen (Populus tremuloides Michx. × Populus tremula L.) Shows Intraspecific Reactions to Illumination In Vitro
Source: Plants (Basel). 2022 Apr 18;11(8):1097. doi: 10.3390/plants11081097 (PMC9024933; doi:10.3390/plants11081097)
Supplement: Supplementary file 1 [file plants-11-01097-s001.zip › plants-1685473-supplementary.pdf]

Table S1

Overall mean (± standard deviation) values of stomata parameters, chloroplast count, leaf thickness and stem anatomical parameters of silver birch and hybrid aspen *in vitro* cultures grown under different spectral compositions.

Silver birch	FL	RGBYO	RB	RGB
stomata, count per mm^2	315 ± 88^{b}	372 ± 54^{a}	294 ± 54^{b}	322 ± 89^{b}
length of stomata, µm	33.2 ± 4.6^{a}	32.3 ± 5.7^{a}	32.0 ± 5.2^{a}	32.8 ± 5.1^{a}
width of stomata, µm	10.8 ± 1.6^{a}	10.8 ± 1.7^{a}	10.9 ± 1.6^{a}	11.1 ± 1.5^{a}
chloroplasts, count	8.2 ± 1.9^{a}	8.5 ± 1.7^{a}	8.1 ± 1.6^{a}	8.1 ± 1.7^{a}
leaf thickness, µm	91.8 ± 12.8^{a}	92.8 ± 12.8^{a}	91.5 ± 11.6^{a}	95.7 ± 13.6^{a}
stem radius, µm	325 ± 75^{a}	334 ± 65^{a}	329 ± 87^{a}	336 ± 87^{a}
xylem width, µm	81.3 ± 25.1^{a}	84.8 ± 25.5^{a}	92.8 ± 40.9^{a}	89.4 ± 40.9^{a}
phloem width, µm	15.9 ± 3.3^{ab}	14.9 ± 2.9^{ab}	16.8 ± 5.7^{a}	16.7 ± 4.9^{a}
secondary xylem cell wall thickness, µm	1.13 ± 0.22^{a}	1.14 ± 0.24^{a}	1.10 ± 0.20^{ab}	1.01 ± 0.23^{b}

Hybrid aspen	FL	RGBYO	RB	RGB
stomata count per mm^2	226 ± 54^{a}	216 ± 60^{a}	211 ± 59^{a}	228 ± 55^{a}
length of stomata, µm	30.5 ± 3.9^{b}	31.4 ± 4.3^{ab}	30.5 ± 4.5^{b}	32.1 ± 4.8^{a}
width of stomata, µm	8.7 ± 0.9^{a}	8.9 ± 1.0^{a}	8.8 ± 1.0^{a}	8.9 ± 1.1^{a}
chloroplasts, count	7.0 ± 1.9^{a}	7.1 ± 1.8^{a}	7.1 ± 1.7^{a}	6.7 ± 1.6^{a}
leaf thickness, µm	96.3 ± 13.7^{a}	98.4 ± 12.7^{a}	98 ± 12.7^{a}	96.5 ± 11.7^{a}
stem radius, µm	314 ± 25^{b}	346 ± 36^{a}	333 ± 38^{ab}	320 ± 34^{b}
xylem width, µm	49.3 ± 14.7^{a}	56.5 ± 16.5^{a}	51.4 ± 11.7^{a}	53.6 ± 13.9^{a}
phloem width, µm	18.6 ± 4.6^{b}	21.2 ± 5.1^{a}	19.7 ± 4.4^{ab}	20.0 ± 4.2^{ab}
secondary xylem cell wall thickness, µm	1.09 ± 0.21^{a}	1.03 ± 0.24^{a}	1.02 ± 0.28^{a}	0.98 ± 0.18^{a}

FL – fluorescent tubes; RGBYO – red+ green+ blue+ yellow+ orange+ far-red LEDs; RB – red+ blue+ far-red LEDs; RGB – red+ green+ blue+ far-red LEDs. Mean (± standard deviation) are shown. Different letters denote significant differences in estimated marginal mean values among the treatments of each parameter at $p<0.05$ according to Tukey's HSD test.

Table S2

Pearson correlation coefficients among the anatomical proxies of hybrid aspen and silver birch by clone across the studied light treatments at jar level.

Silver birch clone No. 40-7	Stomata length	Stomata width	Number of chloroplast	Leaf thickness	Stomatal density	Stem radius	Xylem width	Phloem width
Stomata width	0.77							
Number of chloroplast	0.04	0.06						
Leaf thickness	0.00	-0.01	0.12					
Stomatal density	0.13	0.27	0.25	0.03				
Stem radius	0.05	-0.05	0.14	-0.39	-0.20			
Xylem width	0.27	0.16	0.09	-0.19	-0.07	0.88		
Phloem width	0.15	0.13	0.17	-0.37	-0.07	0.94	0.90	
SXCWT	0.00	-0.15	-0.44	0.04	-0.42	0.27	0.45	0.27

Silver birch clone No. 54-257	Stomata length	Stomata width	Number of chloroplast	Leaf thickness	Stomatal density	Stem radius	Xylem width	Phloem width
Stomata width	0.66							
Number of chloroplast	0.33	0.53						
Leaf thickness	0.06	-0.27	-0.01					
Stomatal density	-0.39	-0.33	-0.29	-0.23				
Stem radius	0.30	0.22	0.00	0.18	-0.19			
Xylem width	0.32	0.09	0.04	0.23	-0.40	0.57		
Phloem width	0.40	0.49	0.50	0.16	-0.47	0.56	0.69	
SXCWT	0.46	0.49	0.43	-0.02	-0.24	0.16	0.31	0.50

Silver birch clone No. Pr33	Stomata length	Stomata width	Number of chloroplast	Leaf thickness	Stomatal density	Stem radius	Xylem width	Phloem width
Stomata width	0.34							
Number of chloroplast	0.04	-0.23						
Leaf thickness	0.24	0.33	-0.59					
Stomatal density	-0.09	-0.16	0.09	0.08				
Stem radius	0.09	0.25	0.15	-0.37	-0.03			
Xylem width	0.04	0.25	0.21	-0.50	-0.11	0.81		
Phloem width	-0.01	0.22	0.26	-0.51	-0.27	0.83	0.90	
SXCWT	-0.16	-0.27	0.12	-0.10	0.20	-0.14	0.21	0.06

Table S2 continued

Hybrid aspen clone No. 5	Stomata length	Stomata width	Number of chloroplast	Leaf thickness	Stomatal density	Stem radius	Xylem width	Phloem width
Stomata width	-0.08							
Number of chloroplast	-0.05	0.11						
Leaf thickness	-0.33	-0.28	0.04					
Stomatal density	-0.18	-0.04	0.18	0.23				
Stem radius	0.36	0.14	-0.34	-0.28	-0.42			
Xylem width	0.08	0.18	-0.28	-0.15	0.07	0.62		
Phloem width	0.47	0.49	-0.18	-0.07	-0.15	0.60	0.57	
SXCWT	-0.42	-0.23	0.75	0.31	0.60	-0.32	-0.08	-0.27

Hybrid aspen clone No. 28	Stomata length	Stomata width	Number of chloroplast	Leaf thickness	Stomatal density	Stem radius	Xylem width	Phloem width
Stomata width	0.38							
Number of chloroplast	0.08	-0.35						
Leaf thickness	0.07	-0.14	-0.11					
Stomatal density	-0.38	-0.44	0.16	0.12				
Stem radius	0.13	-0.28	0.02	-0.01	0.10			
Xylem width	0.30	0.00	0.28	0.16	-0.22	0.13		
Phloem width	0.00	-0.25	0.49	-0.18	0.11	0.38	0.45	
SXCWT	0.04	-0.42	0.08	-0.04	-0.10	0.46	0.03	0.20

Hybrid aspen clone No. 90	Stomata length	Stomata width	Number of chloroplast	Leaf thickness	Stomatal density	Stem radius	Xylem width	Phloem width
Stomata width	0.09							
Number of chloroplast	-0.28	-0.39						
Leaf thickness	0.25	0.27	-0.10					
Stomatal density	0.56	0.08	-0.10	0.37				
Stem radius	0.04	0.22	-0.23	0.43	-0.09			
Xylem width	0.49	0.08	-0.48	0.41	0.35	0.61		
Phloem width	-0.04	-0.01	-0.25	0.14	-0.10	0.50	0.41	
SXCWT	-0.03	-0.08	-0.07	-0.01	-0.07	0.29	0.28	-0.10

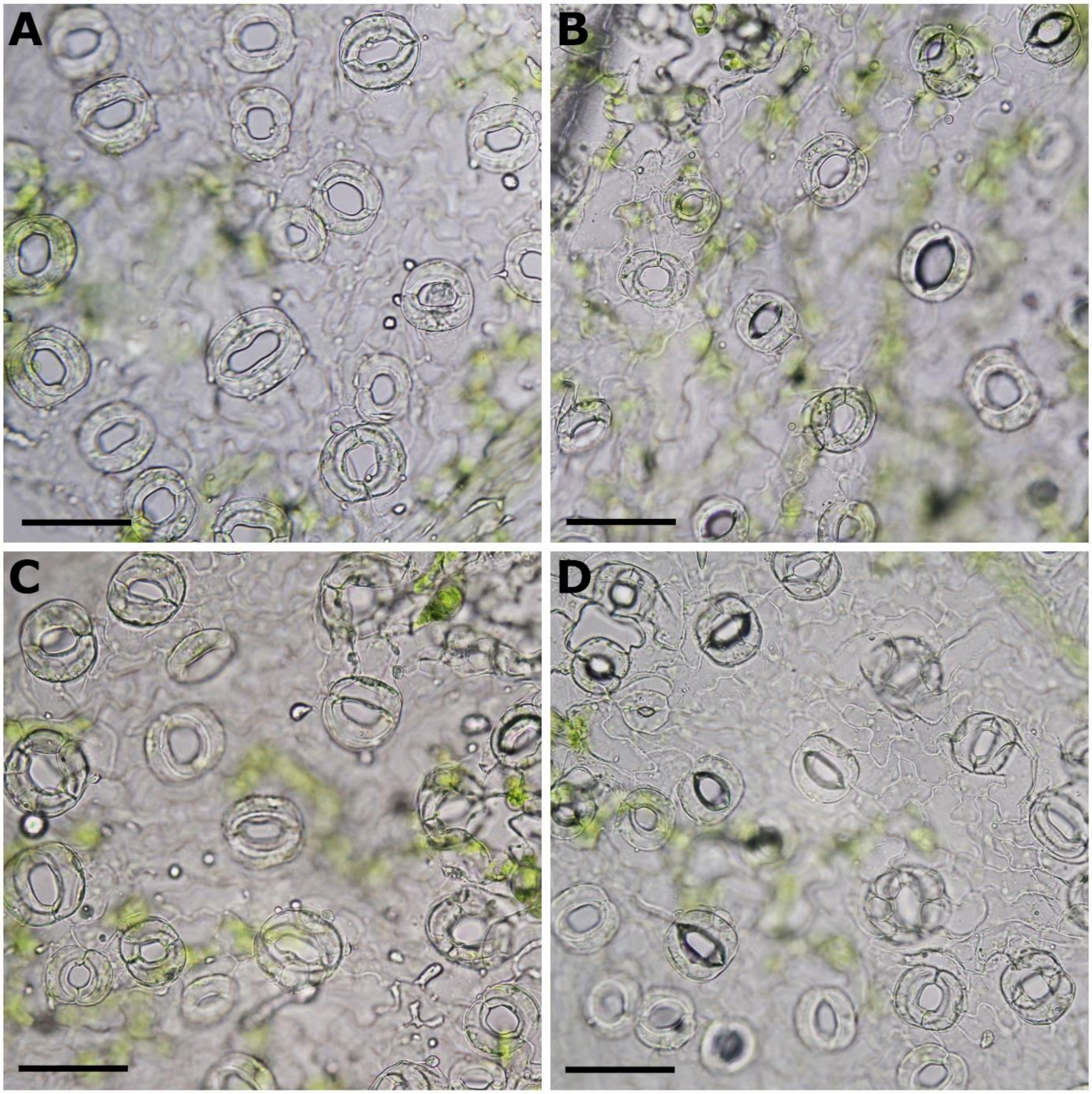

Figure S1. Stomata of silver birch plants grown under different light treatments. FL (A), RB (B), RGB (C) and RGBYO (D). Length of scale cut-off is 50 µm.

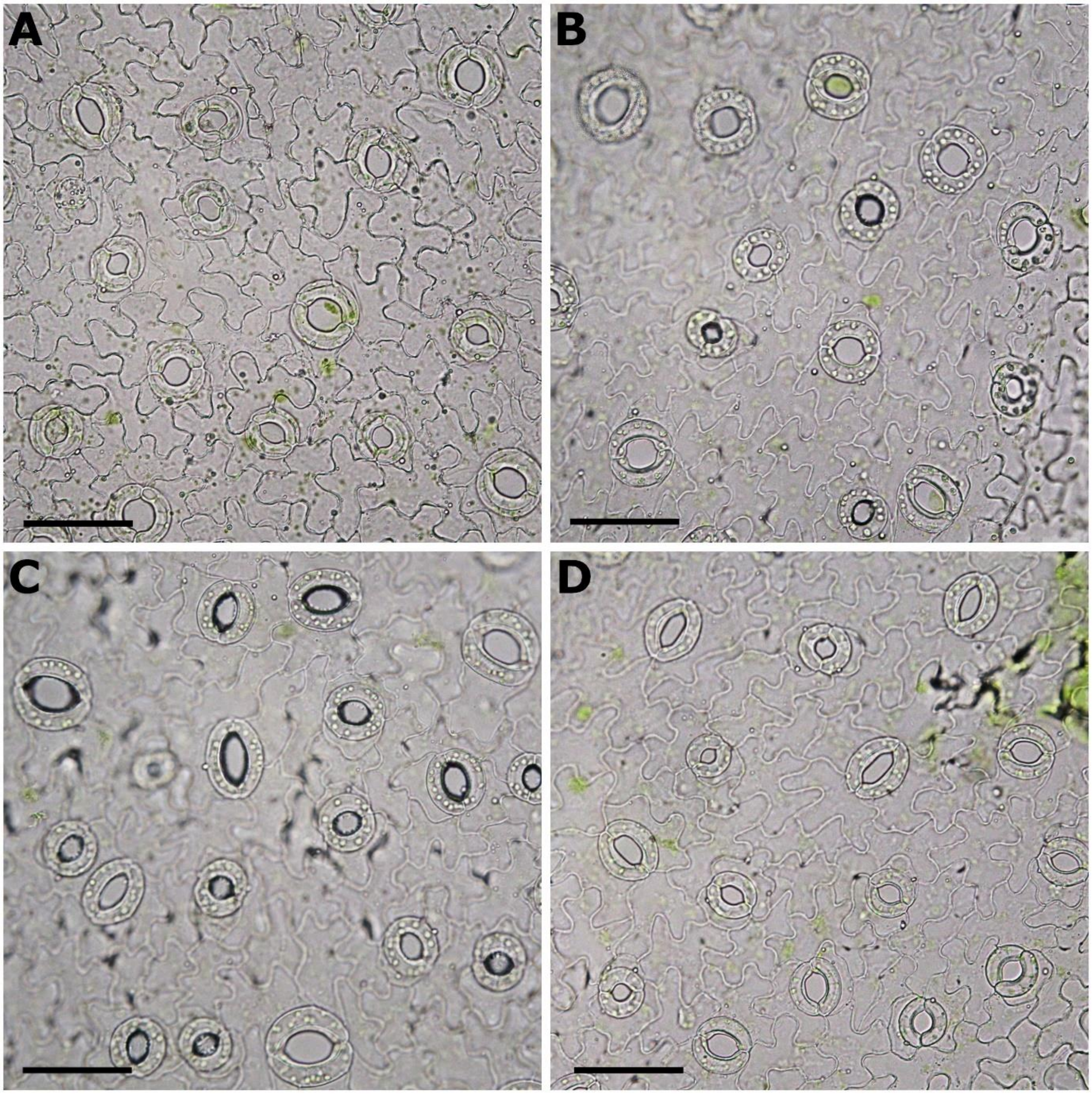

Figure S2. Stomata of hybrid aspen plants grown under different light treatments. FL (A), RB (B), RGB (C) and RGBYO (D). Length of scale cut-off is 50 µm.

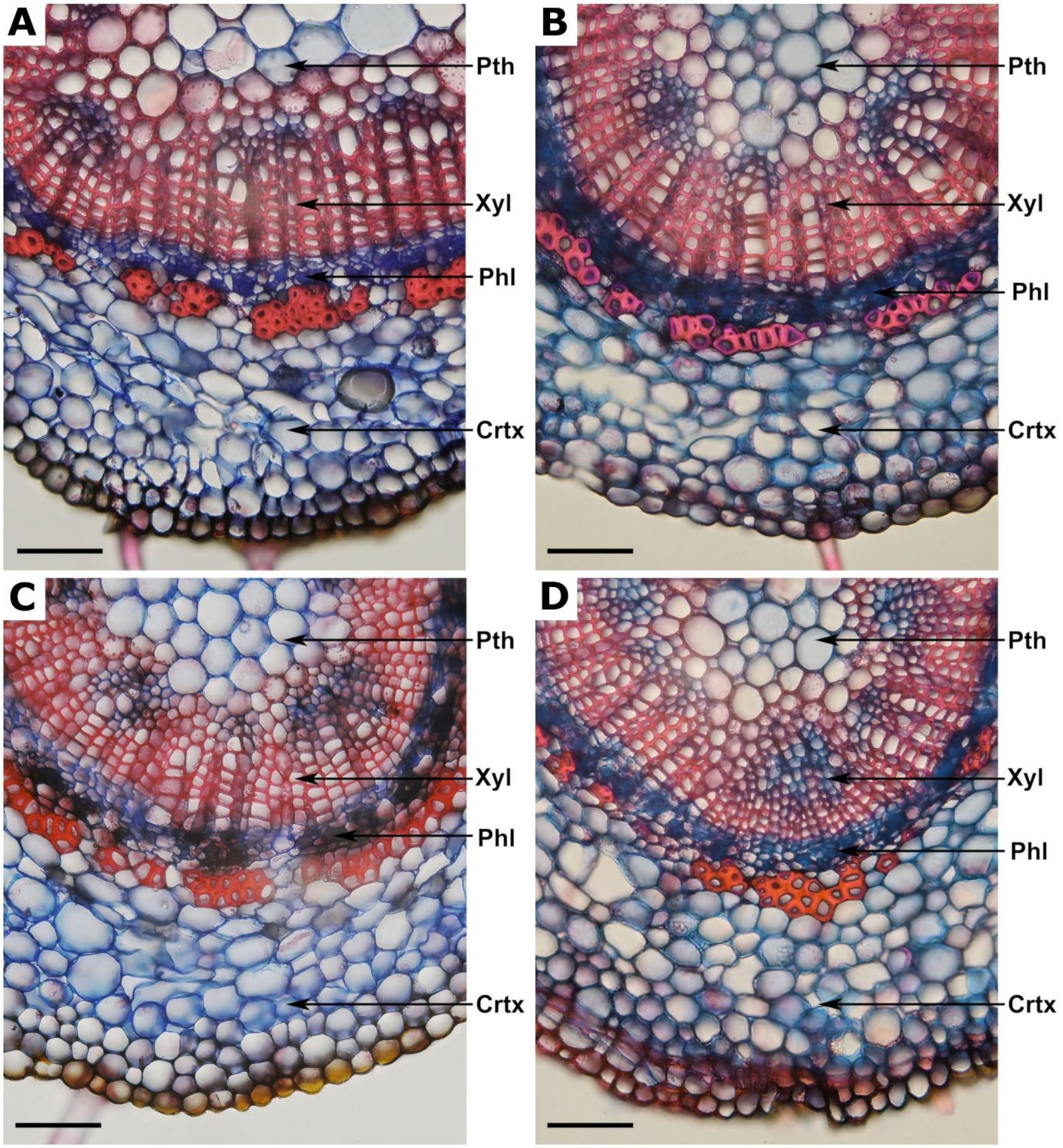

Figure S3. Cross-sections of silver birch *in vitro* plants grown under different light treatments. FL (A), RB (B), RGB (C) and RGBYO (D). Length of scale cut-off is 50 µm. Abbreviations: Pth – pith; Xyl – xylem; Phl – phloem; Crtx – cortex.

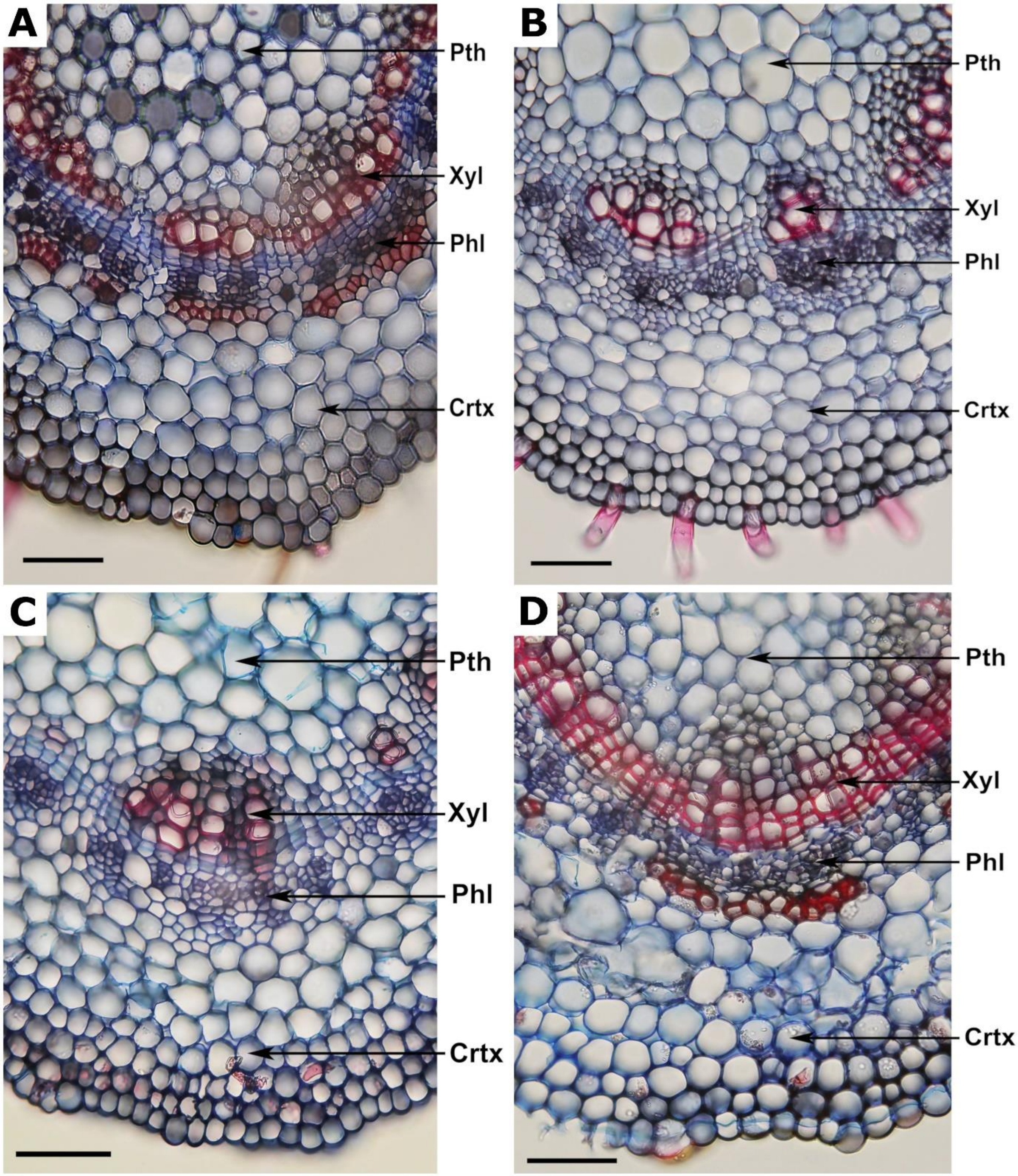

Figure S4. Cross-sections of hybrid aspen *in vitro* plants grown under different light treatments. FL (A), RB (B), RGB (C) and RGBYO (D). Length of scale cut-off is 50 µm. Abbreviations: Pth – pith; Xyl – xylem; Phl – phloem; Crtx – cortex.
